# Rubrene endoperoxide acetone monosolvate

**DOI:** 10.1107/S1600536812008835

**Published:** 2012-03-10

**Authors:** Kiyoaki Shinashi, Akira Uchida

**Affiliations:** a Department of Law, Faculty of Law, Chuogakuin University, Kujike 451, Abiko, Chiba 270-1196, Japan; b Department of Biomolecular Science, Faculty of Science, Toho University, 2-2-1 Miyama, Funabashi, Chiba 274-8510, Japan

## Abstract

The title acetone solvate, C_42_H_28_O_2_·C_3_H_6_O [systematic name: 1,3,10,12-tetra­phenyl-19,20-dioxapenta­cyclo­[10.6.2.0^2,11^.0^4,9^.0^13,18^]icosa-2(11),3,5,7,9,13,15,17-octa­ene acetone monosolvate], is a photooxygenation product of rubrene (systematic name: 5,6,11,12-tetra­phenyl­tetra­cene). The mol­ecule bends at the bridgehead atoms, which are linked by the O—O transannular bond, with a dihedral angle of 49.21 (6)° between the benzene ring and the naphthalene ring system of the tetra­cene unit. In the crystal, the rubrene mol­ecules are linked by C—H⋯O hydrogen bonds into a column along the *c* axis. The acetone solvent mol­ecules form a dimer around a crystallographic inversion centre through a carbon­yl–carbonyl dipolar inter­action. A C—H⋯O hydrogen bond between the rubrene and acetone mol­ecules is also observed.

## Related literature

For related structures, see: Brown & Ehrenberg (1984 ▶); Izuoka *et al.* (1997 ▶); Schuster *et al.* (2002 ▶); Usman *et al.* (2003 ▶); Wang (2008 ▶). For background to photooxygenation of polycyclic aromatic hydro­carbons, see: Sakai *et al.* (1995 ▶).
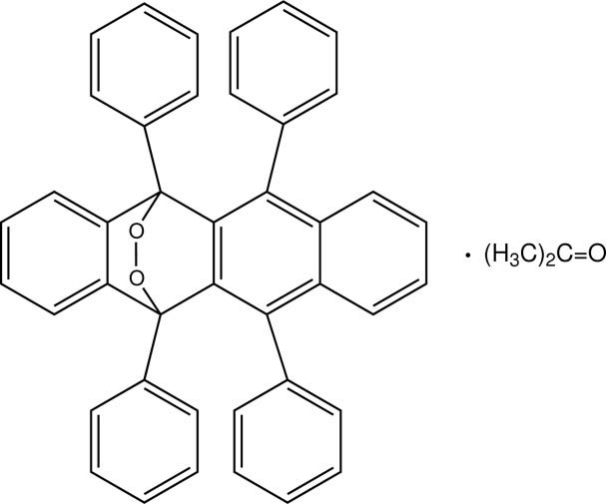


## Experimental

### 

#### Crystal data

C_42_H_28_O_2_·C_3_H_6_O
*M* *_r_* = 622.72
Monoclinic, 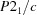

*a* = 11.1592 (6) Å
*b* = 21.2248 (11) Å
*c* = 13.6121 (7) Å
β = 103.113 (1)°
*V* = 3140.0 (3) Å^3^
*Z* = 4
Mo *K*α radiation
μ = 0.08 mm^−1^
*T* = 90 K
0.15 × 0.07 × 0.05 mm

#### Data collection

Bruker APEXII CCD area-detector diffractometer
Absorption correction: multi-scan (*SADABS*; Sheldrick, 1996 ▶) *T*_min_ = 0.988, *T*_max_ = 0.996
20867 measured reflections
7642 independent reflections
5257 reflections with *I* > 2σ(*I*)
*R*_int_ = 0.046

#### Refinement

*R*[*F* ^2^ > 2σ(*F* ^2^)] = 0.049
*wR*(*F* ^2^) = 0.119
*S* = 1.03
7642 reflections
435 parameters
H-atom parameters constrained
Δρ_max_ = 0.39 e Å^−3^
Δρ_min_ = −0.29 e Å^−3^

### 

Data collection: *APEX2* (Bruker, 2007 ▶); cell refinement: *SAINT-Plus* (Bruker, 2007 ▶); data reduction: *SAINT-Plus*; program(s) used to solve structure: *SHELXS97* (Sheldrick, 2008 ▶); program(s) used to refine structure: *SHELXL97* (Sheldrick, 2008 ▶); molecular graphics: *PLATON* (Spek, 2009 ▶) and *ORTEPIII* (Burnett & Johnson, 1996 ▶); software used to prepare material for publication: *SHELXL97* and *PLATON*.

## Supplementary Material

Crystal structure: contains datablock(s) global, I. DOI: 10.1107/S1600536812008835/is5077sup1.cif

Structure factors: contains datablock(s) I. DOI: 10.1107/S1600536812008835/is5077Isup2.hkl

Supplementary material file. DOI: 10.1107/S1600536812008835/is5077Isup3.mol

Supplementary material file. DOI: 10.1107/S1600536812008835/is5077Isup4.cml

Additional supplementary materials:  crystallographic information; 3D view; checkCIF report

## Figures and Tables

**Table 1:** Hydrogen-bond geometry (Å, °)

*D*—H⋯*A*	*D*—H	H⋯*A*	*D*⋯*A*	*D*—H⋯*A*
C28—H28⋯O3^i^	0.95	2.53	3.437 (2)	159
C35—H35⋯O1^ii^	0.95	2.59	3.408 (2)	144

Symmetry codes: (i) 
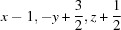
; (ii) 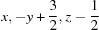.

**Fig. 1.**
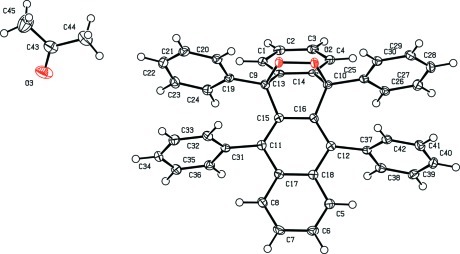
The molecular structure of the title compound, showing displacement ellipsoids at the 50% probability level.

**Fig. 2.**
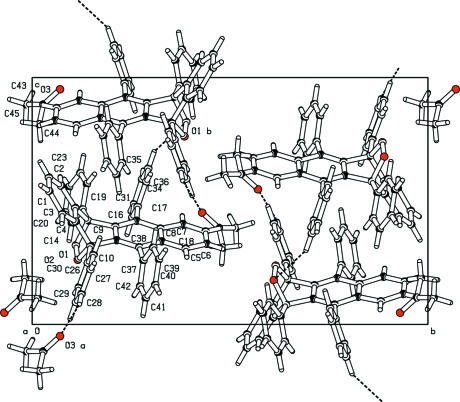
The crystal packing of the title compound viewed along the a axis. The dashed lines indicate C—H···O intermolecular interactions.

## supplementary crystallographic information

### Comment

Polycyclic aromatic hydrocarbons (PAHs) in solution are known to react with molecular oxygen to form endoperoxides when PAHs are irradiated by light with their absorption wavelength. The photooxygenation of PAHs occur due to 1,4-cycloaddition of singlet oxygen to the ground state of a PAH molecule (Sakai *et al.*, 1995). We present here the crystal structure of the title compound.

The molecule bends at the bridgehead atoms, C9 and C10, with a dihedral angle of 49.21 (6)° between the benzene C1–C4/C14/C13 ring and the naphthalene C5–C8/C17/C11/C15/C16/C12/C18 ring system (Figure 1). The adjacent phenyl groups [C19—C24 (centroid *Cg*1), C25—C30 (centroid *Cg*2), C31—C36 (centroid *Cg*3), C37—C42 (centroid *Cg*4)] on either side of the molecule adopt a splayed face-to-face geometry with *Cg*1···*Cg*3 distance of 3.455 (1) Å and *Cg*2···*Cg*4 distance of 3.491 (1) Å (Figure 2). The molecules related by glide plane are linked by C—H···O hydrogen bonds along the *c* axis.

### Experimental

Rubrene was purchased from Sigma-Aldrich. An acetone solution of the compound was exposed to sunlight until the solution became colourless. Crystals of the title compound were obtained from the solution placed in the dark.

### Refinement

All H atoms were placed in geometrical positions and constrained to ride on their parent atoms with C—H = 0.95 for aromatic H atoms and 0.98 Å for CH_3_ type H atoms, respectively. *U*_iso_(H) values were set at 1.2 times *U*_eq_(C) for aromatic H atoms and 1.5 times for methyl H atoms.

### Crystal data

**Table:**

C_42_H_28_O_2_·C_3_H_6_O	*F*(000) = 1312
*M**_r_* = 622.72	*D*_x_ = 1.317 Mg m^−^^3^
Monoclinic, *P*2_1_/*c*	Mo *K*α radiation, λ = 0.71073 Å
Hall symbol: -P 2ybc	Cell parameters from 3033 reflections
*a* = 11.1592 (6) Å	θ = 2.3–26.7°
*b* = 21.2248 (11) Å	µ = 0.08 mm^−^^1^
*c* = 13.6121 (7) Å	*T* = 90 K
β = 103.113 (1)°	Plate, colourless
*V* = 3140.0 (3) Å^3^	0.15 × 0.07 × 0.05 mm
*Z* = 4	

### Data collection

**Table:**

Bruker APEXII CCD area-detector diffractometer	7642 independent reflections
Radiation source: fine-focus sealed tube	5257 reflections with *I* > 2σ(*I*)
Graphite monochromator	*R*_int_ = 0.046
Detector resolution: 8.366 pixels mm^-1^	θ_max_ = 28.1°, θ_min_ = 1.8°
φ and ω scans	*h* = −14→11
Absorption correction: multi-scan (*SADABS*; Sheldrick, 1996)	*k* = −28→23
*T*_min_ = 0.988, *T*_max_ = 0.996	*l* = −17→18
20867 measured reflections	

### Refinement

**Table:**

Refinement on *F*^2^	Primary atom site location: structure-invariant direct methods
Least-squares matrix: full	Secondary atom site location: difference Fourier map
*R*[*F*^2^ > 2σ(*F*^2^)] = 0.049	Hydrogen site location: inferred from neighbouring sites
*wR*(*F*^2^) = 0.119	H-atom parameters constrained
*S* = 1.03	*w* = 1/[σ^2^(*F*_o_^2^) + (0.0487*P*)^2^ + 0.4529*P*] where *P* = (*F*_o_^2^ + 2*F*_c_^2^)/3
7642 reflections	(Δ/σ)_max_ = 0.001
435 parameters	Δρ_max_ = 0.39 e Å^−^^3^
0 restraints	Δρ_min_ = −0.29 e Å^−^^3^

### Special details

**Table:**

Geometry. All e.s.d.'s (except the e.s.d. in the dihedral angle between two l.s. planes) are estimated using the full covariance matrix. The cell e.s.d.'s are taken into account individually in the estimation of e.s.d.'s in distances, angles and torsion angles; correlations between e.s.d.'s in cell parameters are only used when they are defined by crystal symmetry. An approximate (isotropic) treatment of cell e.s.d.'s is used for estimating e.s.d.'s involving l.s. planes.
Refinement. Refinement of *F*^2^ against ALL reflections. The weighted *R*-factor *wR* and goodness of fit *S* are based on *F*^2^, conventional *R*-factors *R* are based on *F*, with *F* set to zero for negative *F*^2^. The threshold expression of *F*^2^ > σ(*F*^2^) is used only for calculating *R*-factors(gt) *etc*. and is not relevant to the choice of reflections for refinement. *R*-factors based on *F*^2^ are statistically about twice as large as those based on *F*, and *R*- factors based on ALL data will be even larger.

### Fractional atomic coordinates and isotropic or equivalent isotropic displacement parameters (Å^2^)

**Table:**

	*x*	*y*	*z*	*U*_iso_*/*U*_eq_	
O1	0.18118 (10)	0.61054 (5)	0.17937 (8)	0.0153 (2)	
O2	0.08624 (10)	0.61524 (5)	0.23817 (8)	0.0159 (2)	
O3	0.56171 (15)	0.92967 (6)	0.04751 (10)	0.0425 (4)	
C1	0.00522 (15)	0.57338 (7)	−0.06128 (11)	0.0157 (3)	
H1	0.0677	0.5707	−0.0983	0.019*	
C2	−0.10563 (15)	0.54225 (7)	−0.09559 (12)	0.0172 (3)	
H2	−0.1206	0.5200	−0.1578	0.021*	
C3	−0.19435 (15)	0.54349 (7)	−0.03941 (12)	0.0179 (3)	
H3	−0.2699	0.5219	−0.0630	0.021*	
C4	−0.17376 (15)	0.57614 (7)	0.05126 (12)	0.0161 (3)	
H4	−0.2333	0.5752	0.0912	0.019*	
C5	0.00268 (15)	0.88782 (7)	0.19322 (12)	0.0174 (3)	
H5	−0.0624	0.8908	0.2274	0.021*	
C6	0.06089 (16)	0.94148 (7)	0.17353 (12)	0.0201 (4)	
H6	0.0358	0.9812	0.1939	0.024*	
C7	0.15725 (16)	0.93803 (7)	0.12347 (12)	0.0219 (4)	
H7	0.1977	0.9754	0.1103	0.026*	
C8	0.19347 (15)	0.88095 (7)	0.09338 (12)	0.0187 (3)	
H8	0.2592	0.8792	0.0597	0.022*	
C9	0.14375 (14)	0.64156 (7)	0.07997 (11)	0.0139 (3)	
C10	−0.02556 (14)	0.64712 (7)	0.18133 (11)	0.0132 (3)	
C11	0.17197 (14)	0.76393 (7)	0.08028 (11)	0.0141 (3)	
C12	−0.02111 (14)	0.77070 (7)	0.18730 (11)	0.0135 (3)	
C13	0.02509 (14)	0.60853 (7)	0.02736 (11)	0.0128 (3)	
C14	−0.06515 (14)	0.61037 (6)	0.08337 (11)	0.0135 (3)	
C15	0.11456 (14)	0.70993 (7)	0.10355 (11)	0.0131 (3)	
C16	0.01819 (14)	0.71332 (7)	0.15861 (11)	0.0131 (3)	
C17	0.13408 (14)	0.82406 (7)	0.11186 (11)	0.0146 (3)	
C18	0.03779 (14)	0.82748 (7)	0.16346 (11)	0.0145 (3)	
C19	0.25208 (14)	0.62739 (7)	0.03296 (12)	0.0146 (3)	
C20	0.35961 (15)	0.60032 (7)	0.08973 (13)	0.0179 (3)	
H20	0.3679	0.5935	0.1599	0.021*	
C21	0.45484 (16)	0.58325 (7)	0.04452 (14)	0.0230 (4)	
H21	0.5275	0.5646	0.0839	0.028*	
C22	0.44413 (16)	0.59327 (8)	−0.05728 (14)	0.0254 (4)	
H22	0.5084	0.5807	−0.0884	0.030*	
C23	0.33927 (16)	0.62178 (8)	−0.11395 (13)	0.0226 (4)	
H23	0.3327	0.6299	−0.1836	0.027*	
C24	0.24402 (15)	0.63844 (7)	−0.06923 (12)	0.0176 (3)	
H24	0.1722	0.6577	−0.1088	0.021*	
C25	−0.11396 (15)	0.64040 (7)	0.25125 (11)	0.0150 (3)	
C26	−0.23815 (15)	0.65513 (7)	0.21634 (12)	0.0163 (3)	
H26	−0.2665	0.6708	0.1497	0.020*	
C27	−0.32065 (15)	0.64729 (7)	0.27750 (12)	0.0204 (4)	
H27	−0.4051	0.6572	0.2525	0.024*	
C28	−0.28005 (16)	0.62495 (7)	0.37559 (13)	0.0220 (4)	
H28	−0.3364	0.6196	0.4178	0.026*	
C29	−0.15715 (16)	0.61064 (7)	0.41095 (13)	0.0208 (4)	
H29	−0.1289	0.5957	0.4780	0.025*	
C30	−0.07414 (15)	0.61793 (7)	0.34927 (12)	0.0173 (3)	
H30	0.0101	0.6075	0.3742	0.021*	
C31	0.27087 (14)	0.76655 (7)	0.02205 (12)	0.0147 (3)	
C32	0.39281 (14)	0.75275 (7)	0.06683 (12)	0.0167 (3)	
H32	0.4146	0.7399	0.1354	0.020*	
C33	0.48295 (15)	0.75768 (7)	0.01173 (13)	0.0208 (4)	
H33	0.5658	0.7473	0.0424	0.025*	
C34	0.45275 (16)	0.77769 (7)	−0.08750 (13)	0.0218 (4)	
H34	0.5146	0.7806	−0.1251	0.026*	
C35	0.33226 (16)	0.79350 (7)	−0.13213 (13)	0.0207 (4)	
H35	0.3116	0.8081	−0.1999	0.025*	
C36	0.24205 (15)	0.78793 (7)	−0.07754 (12)	0.0174 (3)	
H36	0.1595	0.7988	−0.1083	0.021*	
C37	−0.12262 (14)	0.78122 (7)	0.24124 (11)	0.0144 (3)	
C38	−0.23453 (15)	0.80595 (7)	0.18777 (12)	0.0178 (3)	
H38	−0.2470	0.8129	0.1172	0.021*	
C39	−0.32743 (15)	0.82053 (7)	0.23616 (13)	0.0217 (4)	
H39	−0.4033	0.8370	0.1988	0.026*	
C40	−0.30963 (16)	0.81102 (7)	0.33911 (14)	0.0224 (4)	
H40	−0.3733	0.8210	0.3725	0.027*	
C41	−0.19894 (16)	0.78702 (7)	0.39326 (13)	0.0205 (4)	
H41	−0.1871	0.7802	0.4638	0.025*	
C42	−0.10512 (15)	0.77285 (7)	0.34508 (12)	0.0159 (3)	
H42	−0.0287	0.7574	0.3831	0.019*	
C43	0.59016 (18)	0.97168 (8)	0.10896 (13)	0.0279 (4)	
C44	0.5198 (2)	0.98239 (9)	0.18860 (15)	0.0382 (5)	
H44A	0.4545	0.9507	0.1822	0.057*	
H44B	0.4831	1.0246	0.1806	0.057*	
H44C	0.5755	0.9789	0.2553	0.057*	
C45	0.6960 (2)	1.01456 (11)	0.10861 (17)	0.0483 (6)	
H45A	0.7428	0.9987	0.0610	0.072*	
H45B	0.7495	1.0162	0.1764	0.072*	
H45C	0.6651	1.0569	0.0882	0.072*	

### Atomic displacement parameters (Å^2^)

**Table:**

	*U*^11^	*U*^22^	*U*^33^	*U*^12^	*U*^13^	*U*^23^
O1	0.0139 (6)	0.0193 (6)	0.0138 (5)	0.0032 (4)	0.0054 (5)	0.0022 (4)
O2	0.0144 (6)	0.0206 (6)	0.0140 (5)	0.0026 (4)	0.0060 (5)	0.0033 (4)
O3	0.0684 (11)	0.0293 (7)	0.0296 (8)	0.0031 (7)	0.0111 (8)	−0.0067 (6)
C1	0.0177 (8)	0.0144 (7)	0.0154 (8)	0.0020 (6)	0.0049 (7)	0.0025 (6)
C2	0.0223 (9)	0.0141 (7)	0.0136 (8)	0.0013 (6)	0.0004 (7)	−0.0003 (6)
C3	0.0161 (8)	0.0135 (7)	0.0223 (8)	−0.0017 (6)	0.0006 (7)	0.0006 (6)
C4	0.0152 (8)	0.0138 (7)	0.0194 (8)	0.0001 (6)	0.0042 (7)	0.0005 (6)
C5	0.0206 (8)	0.0174 (8)	0.0141 (8)	0.0005 (6)	0.0039 (7)	−0.0003 (6)
C6	0.0268 (9)	0.0138 (8)	0.0197 (8)	−0.0006 (7)	0.0054 (7)	−0.0019 (6)
C7	0.0292 (10)	0.0134 (8)	0.0248 (9)	−0.0050 (7)	0.0093 (8)	0.0004 (6)
C8	0.0217 (9)	0.0179 (8)	0.0184 (8)	−0.0028 (7)	0.0083 (7)	0.0009 (6)
C9	0.0150 (8)	0.0149 (7)	0.0120 (7)	0.0001 (6)	0.0031 (6)	0.0007 (6)
C10	0.0120 (7)	0.0120 (7)	0.0150 (8)	0.0007 (6)	0.0021 (6)	0.0009 (6)
C11	0.0130 (8)	0.0167 (7)	0.0120 (7)	0.0001 (6)	0.0014 (6)	0.0006 (6)
C12	0.0137 (8)	0.0154 (7)	0.0103 (7)	0.0004 (6)	0.0004 (6)	0.0001 (6)
C13	0.0135 (8)	0.0100 (7)	0.0139 (8)	0.0018 (6)	0.0009 (6)	0.0029 (6)
C14	0.0164 (8)	0.0088 (7)	0.0149 (8)	0.0017 (6)	0.0029 (6)	0.0014 (6)
C15	0.0130 (7)	0.0140 (7)	0.0111 (7)	0.0006 (6)	0.0004 (6)	−0.0002 (6)
C16	0.0130 (8)	0.0153 (7)	0.0106 (7)	−0.0013 (6)	0.0017 (6)	0.0013 (6)
C17	0.0166 (8)	0.0151 (7)	0.0115 (7)	−0.0011 (6)	0.0022 (6)	−0.0003 (6)
C18	0.0163 (8)	0.0151 (7)	0.0112 (7)	−0.0003 (6)	0.0015 (6)	−0.0005 (6)
C19	0.0154 (8)	0.0103 (7)	0.0184 (8)	−0.0014 (6)	0.0041 (7)	−0.0033 (6)
C20	0.0169 (8)	0.0150 (8)	0.0220 (9)	−0.0016 (6)	0.0049 (7)	−0.0002 (6)
C21	0.0176 (9)	0.0169 (8)	0.0356 (10)	0.0006 (7)	0.0085 (8)	0.0003 (7)
C22	0.0228 (9)	0.0191 (8)	0.0389 (11)	−0.0024 (7)	0.0167 (9)	−0.0066 (7)
C23	0.0259 (10)	0.0213 (8)	0.0235 (9)	−0.0069 (7)	0.0117 (8)	−0.0048 (7)
C24	0.0177 (8)	0.0163 (8)	0.0189 (8)	−0.0026 (6)	0.0044 (7)	−0.0037 (6)
C25	0.0191 (8)	0.0100 (7)	0.0172 (8)	−0.0025 (6)	0.0067 (7)	−0.0024 (6)
C26	0.0190 (8)	0.0130 (7)	0.0176 (8)	−0.0034 (6)	0.0054 (7)	−0.0013 (6)
C27	0.0180 (9)	0.0184 (8)	0.0261 (9)	−0.0034 (7)	0.0076 (7)	−0.0050 (7)
C28	0.0276 (10)	0.0185 (8)	0.0244 (9)	−0.0049 (7)	0.0155 (8)	−0.0045 (7)
C29	0.0302 (10)	0.0152 (8)	0.0191 (9)	−0.0031 (7)	0.0101 (8)	0.0001 (6)
C30	0.0200 (9)	0.0140 (7)	0.0186 (8)	−0.0008 (6)	0.0058 (7)	0.0000 (6)
C31	0.0163 (8)	0.0107 (7)	0.0178 (8)	−0.0022 (6)	0.0051 (7)	−0.0012 (6)
C32	0.0172 (8)	0.0164 (8)	0.0157 (8)	−0.0027 (6)	0.0024 (7)	−0.0003 (6)
C33	0.0139 (8)	0.0184 (8)	0.0297 (10)	−0.0003 (6)	0.0041 (7)	0.0003 (7)
C34	0.0225 (9)	0.0187 (8)	0.0286 (10)	0.0000 (7)	0.0150 (8)	0.0030 (7)
C35	0.0262 (9)	0.0192 (8)	0.0189 (8)	0.0023 (7)	0.0095 (7)	0.0042 (6)
C36	0.0168 (8)	0.0165 (8)	0.0185 (8)	0.0008 (6)	0.0029 (7)	0.0020 (6)
C37	0.0167 (8)	0.0100 (7)	0.0175 (8)	−0.0012 (6)	0.0057 (7)	−0.0020 (6)
C38	0.0176 (8)	0.0159 (8)	0.0191 (8)	−0.0018 (6)	0.0026 (7)	0.0012 (6)
C39	0.0154 (8)	0.0149 (8)	0.0353 (10)	0.0012 (6)	0.0067 (8)	−0.0004 (7)
C40	0.0219 (9)	0.0171 (8)	0.0327 (10)	−0.0037 (7)	0.0155 (8)	−0.0068 (7)
C41	0.0271 (9)	0.0167 (8)	0.0200 (9)	−0.0066 (7)	0.0102 (7)	−0.0040 (6)
C42	0.0177 (8)	0.0124 (7)	0.0175 (8)	−0.0029 (6)	0.0037 (7)	−0.0023 (6)
C43	0.0367 (11)	0.0251 (9)	0.0203 (9)	0.0068 (8)	0.0032 (8)	0.0025 (7)
C44	0.0422 (13)	0.0383 (11)	0.0361 (11)	0.0029 (9)	0.0129 (10)	−0.0065 (9)
C45	0.0463 (14)	0.0614 (15)	0.0359 (12)	−0.0137 (11)	0.0068 (11)	0.0054 (11)

### Geometric parameters (Å, º)

**Table:**

O1—O2	1.4689 (14)	C23—C24	1.385 (2)
O1—C9	1.4772 (18)	C23—H23	0.9500
O2—C10	1.4749 (18)	C24—H24	0.9500
O3—C43	1.214 (2)	C25—C30	1.391 (2)
C1—C2	1.387 (2)	C25—C26	1.395 (2)
C1—C13	1.393 (2)	C26—C27	1.384 (2)
C1—H1	0.9500	C26—H26	0.9500
C2—C3	1.382 (2)	C27—C28	1.392 (2)
C2—H2	0.9500	C27—H27	0.9500
C3—C4	1.388 (2)	C28—C29	1.380 (2)
C3—H3	0.9500	C28—H28	0.9500
C4—C14	1.396 (2)	C29—C30	1.393 (2)
C4—H4	0.9500	C29—H29	0.9500
C5—C6	1.367 (2)	C30—H30	0.9500
C5—C18	1.425 (2)	C31—C32	1.390 (2)
C5—H5	0.9500	C31—C36	1.396 (2)
C6—C7	1.399 (2)	C32—C33	1.388 (2)
C6—H6	0.9500	C32—H32	0.9500
C7—C8	1.369 (2)	C33—C34	1.383 (2)
C7—H7	0.9500	C33—H33	0.9500
C8—C17	1.427 (2)	C34—C35	1.385 (2)
C8—H8	0.9500	C34—H34	0.9500
C9—C19	1.521 (2)	C35—C36	1.385 (2)
C9—C13	1.526 (2)	C35—H35	0.9500
C9—C15	1.537 (2)	C36—H36	0.9500
C10—C14	1.521 (2)	C37—C42	1.394 (2)
C10—C25	1.525 (2)	C37—C38	1.397 (2)
C10—C16	1.542 (2)	C38—C39	1.384 (2)
C11—C15	1.384 (2)	C38—H38	0.9500
C11—C17	1.440 (2)	C39—C40	1.385 (2)
C11—C31	1.499 (2)	C39—H39	0.9500
C12—C16	1.380 (2)	C40—C41	1.384 (2)
C12—C18	1.444 (2)	C40—H40	0.9500
C12—C37	1.500 (2)	C41—C42	1.389 (2)
C13—C14	1.395 (2)	C41—H41	0.9500
C15—C16	1.445 (2)	C42—H42	0.9500
C17—C18	1.413 (2)	C43—C45	1.492 (3)
C19—C24	1.393 (2)	C43—C44	1.493 (2)
C19—C20	1.395 (2)	C44—H44A	0.9800
C20—C21	1.391 (2)	C44—H44B	0.9800
C20—H20	0.9500	C44—H44C	0.9800
C21—C22	1.380 (2)	C45—H45A	0.9800
C21—H21	0.9500	C45—H45B	0.9800
C22—C23	1.386 (2)	C45—H45C	0.9800
C22—H22	0.9500		
			
O2—O1—C9	112.32 (10)	C24—C23—H23	119.9
O1—O2—C10	111.97 (9)	C22—C23—H23	119.9
C2—C1—C13	120.10 (14)	C23—C24—C19	120.90 (16)
C2—C1—H1	120.0	C23—C24—H24	119.6
C13—C1—H1	120.0	C19—C24—H24	119.6
C3—C2—C1	120.07 (14)	C30—C25—C26	118.69 (14)
C3—C2—H2	120.0	C30—C25—C10	121.29 (14)
C1—C2—H2	120.0	C26—C25—C10	119.99 (14)
C2—C3—C4	120.44 (15)	C27—C26—C25	120.88 (15)
C2—C3—H3	119.8	C27—C26—H26	119.6
C4—C3—H3	119.8	C25—C26—H26	119.6
C3—C4—C14	119.67 (14)	C26—C27—C28	120.10 (16)
C3—C4—H4	120.2	C26—C27—H27	119.9
C14—C4—H4	120.2	C28—C27—H27	119.9
C6—C5—C18	121.30 (15)	C29—C28—C27	119.40 (15)
C6—C5—H5	119.4	C29—C28—H28	120.3
C18—C5—H5	119.4	C27—C28—H28	120.3
C5—C6—C7	120.24 (14)	C28—C29—C30	120.62 (16)
C5—C6—H6	119.9	C28—C29—H29	119.7
C7—C6—H6	119.9	C30—C29—H29	119.7
C8—C7—C6	120.24 (14)	C25—C30—C29	120.31 (15)
C8—C7—H7	119.9	C25—C30—H30	119.8
C6—C7—H7	119.9	C29—C30—H30	119.8
C7—C8—C17	121.05 (15)	C32—C31—C36	118.76 (14)
C7—C8—H8	119.5	C32—C31—C11	121.69 (14)
C17—C8—H8	119.5	C36—C31—C11	119.38 (14)
O1—C9—C19	102.17 (12)	C33—C32—C31	120.29 (15)
O1—C9—C13	105.08 (11)	C33—C32—H32	119.9
C19—C9—C13	113.57 (12)	C31—C32—H32	119.9
O1—C9—C15	105.00 (11)	C34—C33—C32	120.33 (16)
C19—C9—C15	120.06 (12)	C34—C33—H33	119.8
C13—C9—C15	109.23 (12)	C32—C33—H33	119.8
O2—C10—C14	105.51 (11)	C33—C34—C35	120.00 (15)
O2—C10—C25	102.69 (11)	C33—C34—H34	120.0
C14—C10—C25	113.50 (12)	C35—C34—H34	120.0
O2—C10—C16	104.75 (11)	C36—C35—C34	119.69 (15)
C14—C10—C16	109.49 (12)	C36—C35—H35	120.2
C25—C10—C16	119.31 (12)	C34—C35—H35	120.2
C15—C11—C17	118.87 (13)	C35—C36—C31	120.87 (15)
C15—C11—C31	125.89 (13)	C35—C36—H36	119.6
C17—C11—C31	115.23 (13)	C31—C36—H36	119.6
C16—C12—C18	118.85 (13)	C42—C37—C38	118.63 (14)
C16—C12—C37	126.41 (13)	C42—C37—C12	122.00 (14)
C18—C12—C37	114.73 (12)	C38—C37—C12	119.08 (14)
C1—C13—C14	119.72 (14)	C39—C38—C37	120.91 (15)
C1—C13—C9	127.11 (13)	C39—C38—H38	119.5
C14—C13—C9	112.83 (13)	C37—C38—H38	119.5
C13—C14—C4	119.85 (14)	C38—C39—C40	119.89 (16)
C13—C14—C10	113.04 (13)	C38—C39—H39	120.1
C4—C14—C10	126.89 (14)	C40—C39—H39	120.1
C11—C15—C16	120.99 (13)	C41—C40—C39	119.89 (15)
C11—C15—C9	127.08 (13)	C41—C40—H40	120.1
C16—C15—C9	111.91 (12)	C39—C40—H40	120.1
C12—C16—C15	120.80 (13)	C40—C41—C42	120.37 (15)
C12—C16—C10	127.73 (13)	C40—C41—H41	119.8
C15—C16—C10	111.43 (12)	C42—C41—H41	119.8
C18—C17—C8	118.69 (13)	C41—C42—C37	120.28 (16)
C18—C17—C11	120.12 (13)	C41—C42—H42	119.9
C8—C17—C11	121.18 (14)	C37—C42—H42	119.9
C17—C18—C5	118.48 (13)	O3—C43—C45	121.95 (18)
C17—C18—C12	120.33 (13)	O3—C43—C44	120.89 (18)
C5—C18—C12	121.18 (14)	C45—C43—C44	117.16 (17)
C24—C19—C20	118.35 (14)	C43—C44—H44A	109.5
C24—C19—C9	120.63 (14)	C43—C44—H44B	109.5
C20—C19—C9	120.95 (14)	H44A—C44—H44B	109.5
C21—C20—C19	120.63 (15)	C43—C44—H44C	109.5
C21—C20—H20	119.7	H44A—C44—H44C	109.5
C19—C20—H20	119.7	H44B—C44—H44C	109.5
C22—C21—C20	120.20 (17)	C43—C45—H45A	109.5
C22—C21—H21	119.9	C43—C45—H45B	109.5
C20—C21—H21	119.9	H45A—C45—H45B	109.5
C21—C22—C23	119.73 (16)	C43—C45—H45C	109.5
C21—C22—H22	120.1	H45A—C45—H45C	109.5
C23—C22—H22	120.1	H45B—C45—H45C	109.5
C24—C23—C22	120.14 (16)		
			
C9—O1—O2—C10	2.79 (14)	C8—C17—C18—C5	1.1 (2)
C13—C1—C2—C3	−3.2 (2)	C11—C17—C18—C5	−179.82 (14)
C1—C2—C3—C4	0.4 (2)	C8—C17—C18—C12	−177.41 (14)
C2—C3—C4—C14	3.1 (2)	C11—C17—C18—C12	1.7 (2)
C18—C5—C6—C7	−0.2 (3)	C6—C5—C18—C17	−0.6 (2)
C5—C6—C7—C8	0.4 (3)	C6—C5—C18—C12	177.93 (15)
C6—C7—C8—C17	0.2 (3)	C16—C12—C18—C17	−0.1 (2)
O2—O1—C9—C19	−177.18 (10)	C37—C12—C18—C17	−179.42 (14)
O2—O1—C9—C13	−58.38 (13)	C16—C12—C18—C5	−178.62 (14)
O2—O1—C9—C15	56.79 (13)	C37—C12—C18—C5	2.1 (2)
O1—O2—C10—C14	55.15 (13)	O1—C9—C19—C24	168.04 (13)
O1—O2—C10—C25	174.28 (10)	C13—C9—C19—C24	55.43 (18)
O1—O2—C10—C16	−60.39 (13)	C15—C9—C19—C24	−76.45 (18)
C2—C1—C13—C14	2.4 (2)	O1—C9—C19—C20	−8.93 (18)
C2—C1—C13—C9	175.14 (14)	C13—C9—C19—C20	−121.53 (15)
O1—C9—C13—C1	−117.38 (15)	C15—C9—C19—C20	106.58 (16)
C19—C9—C13—C1	−6.5 (2)	C24—C19—C20—C21	−1.8 (2)
C15—C9—C13—C1	130.43 (15)	C9—C19—C20—C21	175.27 (13)
O1—C9—C13—C14	55.81 (15)	C19—C20—C21—C22	0.4 (2)
C19—C9—C13—C14	166.65 (12)	C20—C21—C22—C23	1.5 (2)
C15—C9—C13—C14	−56.38 (16)	C21—C22—C23—C24	−1.9 (2)
C1—C13—C14—C4	1.1 (2)	C22—C23—C24—C19	0.5 (2)
C9—C13—C14—C4	−172.63 (13)	C20—C19—C24—C23	1.4 (2)
C1—C13—C14—C10	176.12 (13)	C9—C19—C24—C23	−175.69 (14)
C9—C13—C14—C10	2.37 (17)	O2—C10—C25—C30	9.34 (17)
C3—C4—C14—C13	−3.8 (2)	C14—C10—C25—C30	122.72 (15)
C3—C4—C14—C10	−178.06 (14)	C16—C10—C25—C30	−105.86 (16)
O2—C10—C14—C13	−58.71 (15)	O2—C10—C25—C26	−168.74 (12)
C25—C10—C14—C13	−170.39 (12)	C14—C10—C25—C26	−55.35 (18)
C16—C10—C14—C13	53.53 (16)	C16—C10—C25—C26	76.06 (18)
O2—C10—C14—C4	115.86 (16)	C30—C25—C26—C27	−0.4 (2)
C25—C10—C14—C4	4.2 (2)	C10—C25—C26—C27	177.76 (13)
C16—C10—C14—C4	−131.89 (15)	C25—C26—C27—C28	0.6 (2)
C17—C11—C15—C16	0.0 (2)	C26—C27—C28—C29	−0.1 (2)
C31—C11—C15—C16	−178.71 (14)	C27—C28—C29—C30	−0.5 (2)
C17—C11—C15—C9	−177.93 (14)	C26—C25—C30—C29	−0.2 (2)
C31—C11—C15—C9	3.4 (3)	C10—C25—C30—C29	−178.33 (13)
O1—C9—C15—C11	118.96 (16)	C28—C29—C30—C25	0.6 (2)
C19—C9—C15—C11	4.9 (2)	C15—C11—C31—C32	−79.6 (2)
C13—C9—C15—C11	−128.79 (16)	C17—C11—C31—C32	101.72 (17)
O1—C9—C15—C16	−59.09 (15)	C15—C11—C31—C36	105.37 (18)
C19—C9—C15—C16	−173.12 (13)	C17—C11—C31—C36	−73.35 (18)
C13—C9—C15—C16	53.16 (16)	C36—C31—C32—C33	−2.7 (2)
C18—C12—C16—C15	−1.5 (2)	C11—C31—C32—C33	−177.81 (13)
C37—C12—C16—C15	177.73 (14)	C31—C32—C33—C34	1.4 (2)
C18—C12—C16—C10	176.09 (14)	C32—C33—C34—C35	0.6 (2)
C37—C12—C16—C10	−4.7 (3)	C33—C34—C35—C36	−1.3 (2)
C11—C15—C16—C12	1.6 (2)	C34—C35—C36—C31	0.0 (2)
C9—C15—C16—C12	179.76 (13)	C32—C31—C36—C35	2.0 (2)
C11—C15—C16—C10	−176.35 (13)	C11—C31—C36—C35	177.23 (13)
C9—C15—C16—C10	1.84 (17)	C16—C12—C37—C42	79.8 (2)
O2—C10—C16—C12	−120.29 (16)	C18—C12—C37—C42	−100.95 (16)
C14—C10—C16—C12	126.97 (16)	C16—C12—C37—C38	−106.47 (18)
C25—C10—C16—C12	−6.2 (2)	C18—C12—C37—C38	72.76 (18)
O2—C10—C16—C15	57.46 (15)	C42—C37—C38—C39	−1.5 (2)
C14—C10—C16—C15	−55.28 (16)	C12—C37—C38—C39	−175.42 (14)
C25—C10—C16—C15	171.56 (13)	C37—C38—C39—C40	0.5 (2)
C7—C8—C17—C18	−1.0 (2)	C38—C39—C40—C41	0.1 (2)
C7—C8—C17—C11	180.00 (15)	C39—C40—C41—C42	0.5 (2)
C15—C11—C17—C18	−1.5 (2)	C40—C41—C42—C37	−1.6 (2)
C31—C11—C17—C18	177.27 (14)	C38—C37—C42—C41	2.0 (2)
C15—C11—C17—C8	177.49 (15)	C12—C37—C42—C41	175.78 (13)
C31—C11—C17—C8	−3.7 (2)		

### Hydrogen-bond geometry (Å, º)

**Table:**

*D*—H···*A*	*D*—H	H···*A*	*D*···*A*	*D*—H···*A*
C28—H28···O3^i^	0.95	2.53	3.437 (2)	159
C35—H35···O1^ii^	0.95	2.59	3.408 (2)	144

Symmetry codes: (i) *x*−1, −*y*+3/2, *z*+1/2; (ii) *x*, −*y*+3/2, *z*−1/2.
